# Development of Anthraquinone Analogues as Phosphoglycerate Mutase 1 Inhibitors

**DOI:** 10.3390/molecules24050845

**Published:** 2019-02-27

**Authors:** Ke Huang, Lulu Jiang, Huiti Li, Deyong Ye, Lu Zhou

**Affiliations:** Department of Medicinal Chemistry, School of Pharmacy, Fudan University, No. 826, Zhangheng Rd., Shanghai 201203, China; kehuang13@fudan.edu.cn (K.H.); 18111030012@fudan.edu.cn (L.J.); 17211030005@fudan.edu.cn (H.L.)

**Keywords:** phosphoglycerate mutase 1, anthraquinone inhibitors, co-crystal structure, cancer treatment

## Abstract

Phosphoglycerate mutase 1 (PGAM1) coordinates glycolysis and biosynthesis to promote cancer cell proliferation, and is believed to be a promising target for cancer therapy. Herein, based on the anthraquinone scaffold, we synthesized 31 anthraquinone derivatives and investigated the structure−activity relationship (SAR). The 3-substitient of sulfonamide on the anthraquinone scaffold was essential for maintaining potency and the modifications of the hydroxyl of alizarin would cause a sharp decrease in potency. In the meantime, we determined the co-crystal structure of PGAM1 and one of the anthraquinone inhibitors **9i** with IC_50_ value of 0.27 μM. The co-crystal structure revealed that F22, K100 and R116 of PGAM1 were critical residues for the binding of inhibitors which further validated the SAR. Consistent with the crystal structure, a competitive assay illustrated that compound **9i** was a noncompetitive inhibitor. In addition, compound **9i** effectively restrained different lung cancer cells proliferation in vitro. Taken together, this work provides reliable guide for future development of PGAM1 inhibitors and compound **9i** may act as a new leading compound for further optimization.

## 1. Introduction

In 1924, Warburg discovered that cancer cells tended to metabolize glucose through aerobic glycolysis rather than oxidative phosphorylation, even if there was sufficient oxygen [1,2]. This character of cancer cells was distinct from normal differentiated cells and the phenomenon was thereafter named the “Warburg effect”. However, the reason why cancer cells adopt such an altered metabolism remained unclear. In the past decades, reprogramming energy metabolism was considered as an emerging hallmark of cancer cells [3] and the study of cancer metabolism has drawn great interest [4,5,6,7,8]. During aerobic glycolysis of cancer cells, adenosine 5′-triphosphate (ATP) is produced inefficiently in this metabolic pattern, whereas building blocks such as nucleotides, and amino acids are generated in large amounts, satisfying the demands of anabolic biosynthesis which are essential for rapid cell proliferation [9,10,11,12]. Nowadays, various enzymes playing critical roles in cancer metabolism have been reported, including isocitrate dehydrogenase [13,14,15]. Inhibitors targeting these enzymes have been approved by Food and Drug Administration [14,15] which further demonstrated the potential of modulating cancer metabolism for cancer therapy.

Phosphoglycerate mutase 1 (PGAM1) is a key enzyme in the 8th step of glycolysis pathway converting 3-phosphoglycerate (3PG) to 2-phosphoglycerate (2PG) [16]. Previously we found that PGAM1 could balance the intracellular concentrations of its substrate 3PG and its product 2PG to control tumor growth [17]. In detail, 3PG inhibits 6-phosphogluconate dehydrogenase in pentose phosphate pathway (PPP) and 2PG activates 3-phosphoglycerate dehydrogenase in serine synthesis [17]. When PGAM1 is inhibited by a small molecule or shRNA, intracellular level of 3PG increased and 2PG decreased which led to the blockade of PPP, serine synthesis, glycolysis and subsequently impaired tumor growth. Since PGAM1 regulates both glycolysis and biosynthesis, developing inhibitors targeting PGAM1 is considered as a way of dual inhibition of catabolism and anabolism for cancer therapy [18]. Furthermore, PGAM1 is upregulated in many kinds of tumors and the expression of PGAM1 is correlated with cancer patients’ prognosis [19,20,21]. Recently, more functions of PGAM1 in cancer metabolism were described. Posttranslational modulations of PGAM1 such as Y26 phosphorylation [22] and deacetylation of K100 [23] would promote cancer cell proliferation and tumor growth. Besides, it is essential for homologous recombination repair by regulating the deoxyribonucleotide triphosphate nucleoside (dNTP) pool [24] and it facilitates cancer cell migration independent of its metabolic function [25]. Taken together, PGAM1 is a promising target for cancer treatment.

**Figure 1:**
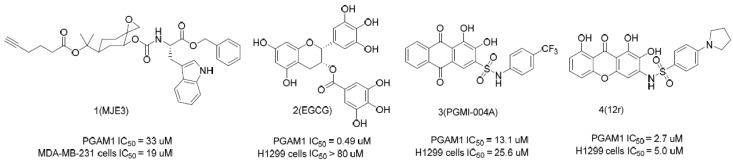
Reported phosphoglycerate mutase 1 (PGAM1) inhibitors.

However, up to now, only four representative PGAM1 inhibitors were reported (Figure 1). The first PGAM1 inhibitor 1 (MJE3) was discovered by the Cravatt group in 2005 [26]. It was identified as a covalent inhibitor labeling the K100 of PGAM1 by spiroepoxides [27]. Another PGAM1 inhibitor described these days was 2((−)-epigallocatechin-3-gallate (EGCG)), a natural product from green tea [28]. It demonstrated strong inhibition activity towards PGAM1 [28] but it was reported to display extensive potency on multiple targets [29,30,31]. We discovered the other two inhibitors, one of which was anthraquinone derivative **3** (PGMI-004A) [17] and the other was the xanthone derivative **4** (12r) [32]. Both of them showed moderate inhibition activity on PGAM1 and cancer cell proliferation.

Since only a limited amount of anthraquinone inhibitors were reported, the structure−activity relationship (SAR) of anthraquinone derivatives and PGAM1 was not well studied yet. Besides, up to these days, the molecular interactions of PGAM1 and anthraquinone inhibitor were not observed in crystal structure. However, anthraquinones exhibit a variety of pharmacological activities including anticancer [33,34,35], antifungal [36], and antibacterial properties [37] and so on. What’s more, there are many clinically approved anthraquinone drugs such as doxorubicin and mitoxantrone with strong potency in suppressing cancer cell proliferation [38]. Here we designed and synthesized a series of new anthraquinone compounds and evaluated their biological activity on PGAM1 and cancer cell proliferation. In addition, we solved the co-crystal structure of PGAM1 and compound **9i** which interpreted the molecular mechanism of compound **9i** interacting with PGAM1 and further confirmed the SAR we investigated. Combined with the crystal structure, additional competitive assay demonstrated the noncompetitive binding of compound **9i** with the 3PG substrate.

## 2. Results and Discussion

### 2.1. Chemistry

**Figure 2:**
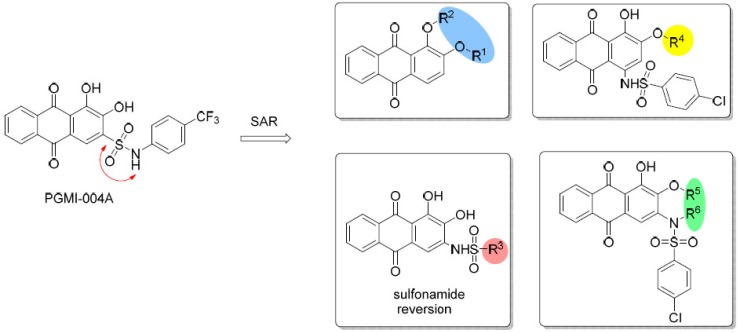
The optimization strategy and SAR exploration of the anthraquinone derivatives against PGAM1.

Four classes of anthraquinone derivatives were synthesized, with 1,2-substituted, 3-substituted, 2,4-substituted, and 2,3-substituted anthraquinone core scaffolds (Figure 2). 

**Scheme 1:**
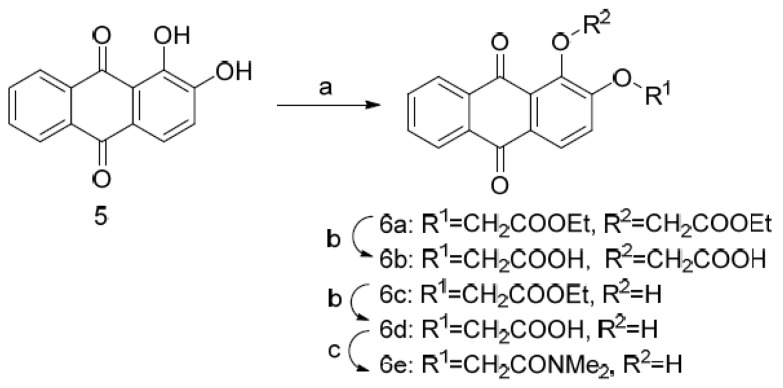
Synthesis of compounds **6a**–**e**. *Reagents and conditions*: (**a**) BrCH_2_COOEt, K_2_CO_3_, DMF, 80 °C; (**b**) LiOH, MeOH, H_2_O, 30 °C; (**c**)NHMe_2_·HCl, EDCI, HOBt, DIPEA, DMF.

As shown in Scheme 1, starting from commercially available alizarin, compounds **6a**–**e** were obtained by substitution with ethyl bromoacetate, hydrolysis of the ester, and amidation.

**Scheme 2:**
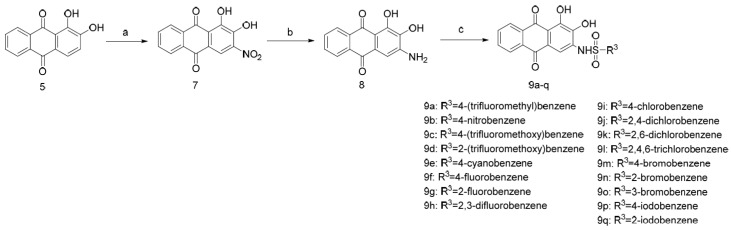
Synthesis of compounds **9a**–**q**. Reagents and conditions: (**a**) HNO_3_, CH_3_COOH, 50 °C; (**b**) Sn, SnCl_2_·H_2_O, HCl, EtOH; (**c**) R^3^SO_2_Cl, Py.

In Scheme 2, firstly, alizarin was nitrated by HNO_3_ and then reduced by Sn and HCl to afford the key amine intermediate **8**, which was reacted with diverse sulfonyl chlorides to give the target compounds **9a**–**q**.

**Scheme 3:**
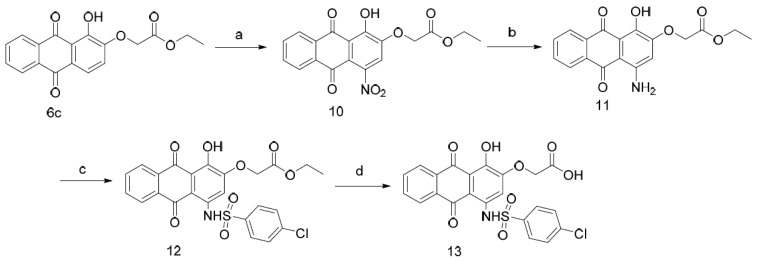
Synthesis of compounds **12** and **13**. Reagents and conditions: (**a**) HNO_3_, CH_3_COOH, 50 °C; (**b**) Sn, SnCl_2_·H_2_O, HCl, EtOH; (**c**) 4-chlorobenzenesulfonyl chloride, Py; (**d**) LiOH, MeOH, H_2_O, 30 °C.

As shown in Scheme 3, the 4-nitro group was reduced to an amino group after the nitrification of compound **6c**, followed by sulfonylation of the amino group and hydrolysis of the ester as described above.

**Scheme 4:**
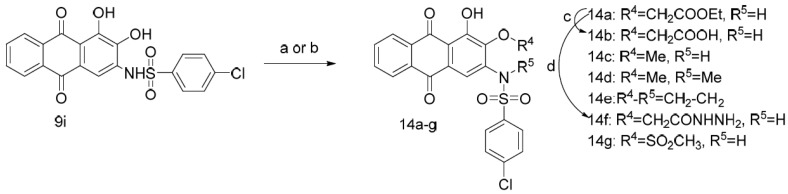
Synthesis of compounds **14a**–**g**. Reagents and conditions: (**a**) K_2_CO_3_, DMF, corresponding substituted reagent; (**b**) methanesulfonyl chloride, DIPEA, Py; (**c**) LiOH, MeOH, H_2_O, 30 °C; (**d**) NH_2_NH_2_·H_2_O, EtOH, reflux.

Finally, compound **9i** was employed as a starting material, and compounds **14a**–**g** were obtained by substitution with different reagents and further hydrolysis of the ester or amidation of the carboxyl group (Scheme 4).

### 2.2. SAR Exploration of the Anthraquinone Derivatives Against PGAM1

Previously we reported several anthraquinone compounds as PGAM1 inhibitors [17]. Due to the limited amount of compounds and the lack of chemical diversity, the SAR of anthraquinone derivatives and PGAM1 remained poorly understood. Here we synthesized four classes of compounds on the base of bioisosterism to study the SAR of anthraquinone derivatives against PGAM1 in detail (Figure 2), using an optimized enzymatic assay in which PGMI-004A served as a control with an IC_50_ value of 1.2 ± 0.3 μM. Firstly, we found that different modifications of the hydroxyl of alizarin (**6a**–**e**) showed weak PGAM1 inhibition activity at 10 μM (Table 1).

In order to explore the SAR of 3-substituents of the anthraquinone scaffold against PGAM1, compounds **9a**–**q** were synthesized by reversing the sulfonamide group of PGMI-004A. Compounds **9a**–**e** with different substituents on the phenyl ring displayed comparable inhibition activity towards PGAM1, with IC_50_ values of 0.5 μM or so (Table 2). They were much more potent than the compounds without the sulfonamide group, suggesting the sulfonamide group was essential for maintaining potency. Then, we studied the impact of substituents of different halogens on the phenyl ring (**9f**–**q**) on the inhibition activity towards PGAM1 (Table 2). Generally, compounds with Cl, Br and I substituents (**9i**–**q**) with IC_50_ values below 0.5 μM performed better than F substituents (**9f**–**h**). Among them, we discovered one of the most potent PGAM1 inhibitors **9i** with an IC_50_ value of 0.27 μM and we solved the co-crystal structure of compound **9i** with PGAM1 which would be discussed below.

We next moved the sulfonamide to the C4 position of alizarin by substituting the hydroxyl, followed by nitrification, reduction, and sulfonylation. Probably due to the modification of the hydroxyl and replacement of the sulfonamide, compounds 12 and 13 failed to maintain potency, decreasing 10% and 34% of PGAM1 activity at 10 μM, respectively. Finally, considering the good solubility and potency of inhibitor **9i**, we kept the 4-chlorobenzene group and synthesized compounds **14a**–**g**. However, diverse modifications of the hydroxyl caused a loss of inhibition activity toward PGAM1 to different extents (Table 3).

In order to exclude the inhibition of the other three enzymes (enolase-pyruvate kinase-LDH) in the coupled assay, we performed counter screening of selected compounds with IC_50_ value below 1 μM on PGAM1.

As shown in Table 4, the compounds displayed almost no inhibition on the three downstream enzymes at 0.5 μM which further confirmed the on-target effect of the compounds.

### 2.3. Binding Mode of Compound ***9i*** with PGAM1

**Figure 3:**
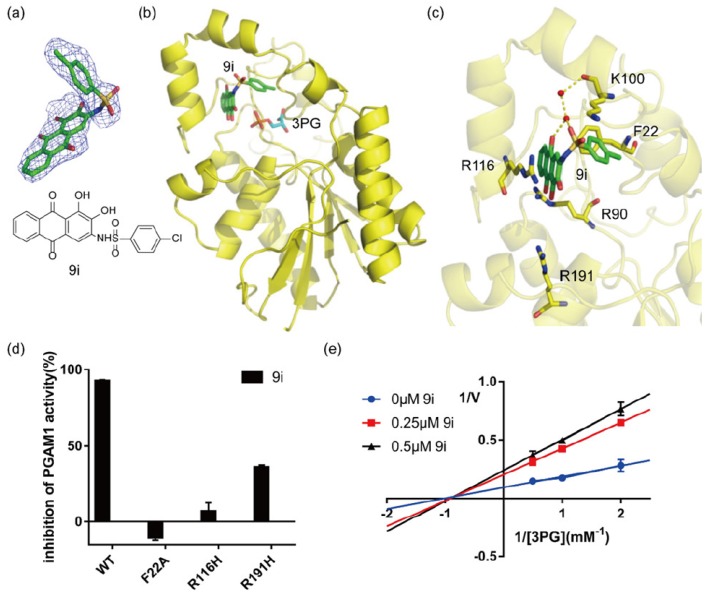
Binding mode of anthraquinone inhibitor **9i** with PGAM1. (**a**) Chemical structure of compound **9i** and Fo–Fc electron density of compound **9i** contoured at 2.0σ; (**b**) Overlay of compound **9i** (PBD: 6ISN) and 3PG (PBD:2F90) in PGAM1; (**c**) Interactions of compound **9i** and the critical residues of PGAM1 in the co-crystal structure; (**d**) Inhibition of compounds **9i** on wild-type and mutations of PGAM1 at concentration of 5 μM; (**e**) Noncompetitive property of compound **9i** with substrate 3PG. The data are presented as mean ±s.d.

To further understand the molecular mechanism of the anthraquinone derivatives interacting with PGAM1, we determined the X-ray structure of PGAM1 in complex with compound **9i** at resolution of 1.98 Å (Table 5). Compound **9i** occupied a novel allosteric site adjacent to substrate binding site with nice electron density (Figure 3A,B). The allosteric pocket was surrounded by the residues of F22, R90, K100, R116 and R191. In detail, the anthraquinone scaffold and sulfonamide of compound **9i** interacted with the main chain carbonyl of K100 through water bridges (Figure 3C). In addition, a hydrophobic interaction was observed between F22 and chlorine-substituted phenyl ring of compound **9i** (Figure 3C). Compound **9i** also engaged in a π-cation interaction with R116 (Figure 3C), which explains why modifications of the hydroxyl group led to decreased potency [39]. To validate the binding mode revealed by the co-crystal structure, we tested the activity of PGAM1 mutants (Supplementary Data, Figure S1) and the inhibition activity of compound **9i** on different mutations of PGAM1. Compound **9i** failed to inhibit mutations of PGAM1 (F22A, R116H and R191H) as effectively as the wild type at concentration of 5 μM which agreed with the results from crystal structure. Furthermore, a substrate competitive assay demonstrated that compound **9i** held a non-competitive property with substrate 3PG which was also consistent with the binding mode revealed by X-ray structure. The co-crystal structure together with the molecular biological assays illustrated the binding mode of the anthraquinone inhibitor with PGAM1 and provided useful information for further optimization.

### 2.4. Inhibition Activity of Selected Compounds on Cancer Cell Proliferation

Given PGAM1 plays a crucial part in cancer metabolism, inhibition of PGAM1 by small molecules is supposed to suppress cancer cell proliferation. We selected the compounds with IC_50_ values below 10 μM toward PGAM1 to inhibit H1299 cells proliferation. Then the potent inhibitors with IC_50_ values below 20 μM on H1299 cells were further tested on A549 and PC9 cells. In general, proliferation inhibition of the compounds was correlated with PGAM1 inhibition and the inhibitors performed similarly among different cancer cells, with IC_50_ values ranging from approximately 6–50 μM (Table 6 and Table 7). Notably, compound **9i** effectively suppressed the three different cancer cells proliferation which was also potent towards PGAM1.

## 3. Materials and Methods

### 3.1. General Procedures

All reagents were purchased commercially. ^1^H-NMR and ^13^C-NMR spectra were recorded on Bruker AC400 and Bruker AC600 NMR spectrometers, respectively (Billerica, MA, USA). Low-resolution mass spectra were recorded on a 6120 Quadrupole mass spectrometer (Agilent, Santa Clara, CA, USA) equipped with electrospray ionization (ESI). High-resolution mass spectra were determined on triple TOF 5600^+^ MS/MS system (AB Sciex, Concord, ON, Canada) in negative ESI mode. The purity of target compounds was determined by high-performance liquid chromatography (Agilent, Santa Clara, CA, USA, DIKMA Diamonsil Plus C18, 250 × 4.6 mm, 5 μm, 25 °C, UV 290 nM). All the biologically tested compounds achieved ≥95% purity.

### 3.2. Synthesis of diethyl 2,2′-((9,10-dioxo-9,10-dihydroanthracene-1,2-diyl) bis(oxy)) diacetate *(**6a**)*

1,2-Dihydroxyanthracene-9,10-dione (1.2 g, 5 mmol), ethyl 2-bromoacetate (2 g, 12 mmol) and K_2_CO_3_ (1.66 g, 12 mmol) were added to *N*,*N*-dimethylformamide (DMF, 50 mL) and the mixture was stirred at 80 °C. The mixture was cooled to room temperature and poured into 10% aqueous HCl (350 mL). Then the suspension was extracted with ethyl acetate and the organic phase was washed with brine, dried over anhydrous Na_2_SO_4_ and filtered. The residue was purified by silica chromatography after removal of ethyl acetate to afford 6a as a yellow solid (0.89 g, 43%). ^1^H-NMR (400 MHz, DMSO-*d*_6_) δ 8.16–8.10 (m, 2H), 8.01 (d, *J* = 8.8 Hz, 1H), 7.93–7.84 (m, 2H), 7.53 (d, *J* = 8.8 Hz, 1H), 5.07 (s, 2H), 4.74 (s, 2H), 4.20 (qd, *J* = 4.0, 7.2 Hz, 4H), 1.23 (td, *J* = 2.4, 7.2 Hz, 6H). ^13^C-NMR (151 MHz, DMSO) δ 181.69, 181.42, 168.27, 167.97, 156.49, 146.23, 134.58, 134.32, 133.94, 132.26, 127.15, 126.94, 126.67, 126.18, 124.64, 118.20, 68.72, 65.22, 60.99, 60.44, 14.07, 13.98. MS (ESI) (*m*/*z*): 413.1 (M − H)^−^. HRMS (ESI) calcd. for C_22_H_20_O_8_ [M − H]^−^: 413.1231; found: 413.1239. Purity 95.1% by HPLC.

### 3.3. Synthesis of 2,2′-((9,10-dioxo-9,10-dihydroanthracene-1,2-diyl) bis(oxy)) diacetic acid *(**6b**)*

Compound **6a** (206 mg, 0.5 mmol) and 0.5M aqueous LiOH (8 mL) were added into methanol (8 mL) and the mixture was stirred at 30 °C. The mixture was added dropwise to 10% aqueous HCl and acidified to pH 1–2. A yellow solid (169 mg, 95%) of 6b was obtained by filtration. ^1^H-NMR (400 MHz, DMSO-*d*_6_) δ 12.76 (brs, 2H), 8.14 (d, *J* = 7.6 Hz, 2H), 8.01 (d, *J* = 8.4 Hz, 1H), 7.93–7.85 (m, 2H), 7.51 (d, *J* = 8.8 Hz, 1H), 4.98 (s, 2H), 4.67 (s, 2H). ^13^C-NMR (151 MHz, DMSO) δ 181.95, 181.45, 169.75, 169.45, 156.76, 146.32, 134.62, 134.31, 133.98, 132.34, 126.97, 126.82, 126.71, 126.20, 124.61, 118.12, 68.62, 65.05. MS (ESI) (*m*/*z*): 355.0 (M − H)^−^. HRMS (ESI) calcd. for C_18_H_12_O_8_ [M − H]^−^: 355.0459; found: 355.0462. Purity 95.1% by HPLC.

### 3.4. Synthesis of 2-((1-hydroxy-9,10-dioxo-9,10-dihydroanthracen-2-yl) oxy)-N,N-dimethylacetamide *(**6e**)*

Compound **6d** (149 mg, 0.5 mmol), dimethylamine hydrochloride (31 mg, 0.68 mmol), *N*-(3-dimethylaminopropyl)-*N′*-ethylcarbodiimide hydrochloride (EDCI, 120 mg, 0.6 mmol), 1-hydroxybenzotriazole (HOBt, 80 mg, 0.6 mmol) and diisopropylethylamine (DIPEA, 175 μL, 1 mmol) were added to dry DMF (10 mL) and the mixture was stirred at room temperature. The mixture was added dropwise to H_2_O (140 mL). An orange solid (114 mg, 70%) of 6e was obtained by filtration. ^1^H-NMR (400 MHz, DMSO-*d*_6_) δ 12.74 (s, 1H), 8.28–8.11 (m, 2H), 7.99–7.87 (m, 2H), 7.68 (d, *J* = 8.4 Hz, 1H), 7.31 (d, *J* = 8.8 Hz, 1H), 5.09 (s, 2H), 3.02 (s, 3H), 2.87 (s, 3H). ^13^C-NMR (151 MHz, DMSO) δ 188.67, 180.76, 166.18, 152.60, 151.79, 135.20, 134.26, 133.48, 132.95, 126.81, 126.60, 124.89, 120.20, 118.29, 115.94, 66.12, 35.46, 35.01. MS (ESI) (*m*/*z*): 324.0 (M − H)^−^. HRMS (ESI) calcd. for C_18_H_15_NO_5_ [M − H]^−^: 324.0877; found: 324.0876. Purity 96.4% by HPLC.

### 3.5. General Procedure for the Synthesis of Compounds ***9a***–***q***

Step 1. Nitric acid (1.5 mL, 33.33 mmol, Sinopharm Chemical Reagent Co., Ltd, Shanghai, China) was added to the suspension of alizarin (5 g, 20.8 mmol) in acetic acid (350 mL) at 50 °C. A yellow solid (4.15 g, 70%) of crude product 6 was obtained by filtration [40].

Step 2. Sn (10.5 g, 341 mmol, Sinopharm Chemical Reagent Co., Ltd, Shanghai, China), SnCl_2_·2H_2_O (12.5 g, 55.4 mmol) and concentrated HCl (50.4 mL, 604.8 mmol) were added to the suspension of crude product 6 (1.75 g, 6.14 mmol) in ethanol (350 mL, Sinopharm) and the mixture was stirred at room temperature overnight. Then the mixture was poured into water (1 L) and a red solid precipitated. A black solid of crude product 7 (1.41 g, 90%) was obtained after filtration and being dried under vacuum [40].

Step 3. To the solution of crude product 7 (255 mg, 1 mmol) in dry pyridine (5 mL, Sinopharm), corresponding sulfonyl chloride (1.5 mmol, Energy Chemical, Shanghai, China) was added and the mixture was stirred at room temperature. Then it was added to 10% aqueous HCl (50 mL, Sinopharm) and the suspension was extracted with ethyl acetate (Sinopharm). The organic phase was washed with brine, dried over anhydrous Na_2_SO_4_ and filtered. The residue was purified by silica chromatography after removal of ethyl acetate to give compounds **9a**–**q**.

*N-(3,4-Dihydroxy-9,10-dioxo-9,10-dihydroanthracen-2-yl)-4-(trifluoromethyl) benzenesulfonamide* (**9a**). Yellow solid, 25% yield. ^1^H-NMR (400 MHz, DMSO-*d*_6_) δ 12.60 (brs, 1H), 10.90 (brs, 1H), 10.67 (brs, 1H), 8.22–8.12 (m, 2H), 8.09 (d, *J* = 8.4 Hz, 2H), 8.01 (d, *J* = 8.4 Hz, 2H), 7.95–7.86 (m, 2H), 7.73 (s, 1H). ^13^C-NMR (151 MHz, DMSO) δ 187.78, 180.56, 150.37, 144.20, 143.23, 135.02, 134.22, 133.28, 132.79, 132.68 (q, *J* = 31.7 Hz), 130.34, 127.60 (2C), 126.77, 126.61, 126.59, 126.39, 123.71, 123.38 (q, *J* = 273.3 Hz), 113.49, 113.35. MS (ESI) (*m*/*z*): 462.0 (M − H)^−^. HRMS (ESI) calcd. for C_21_H_12_F_3_NO_6_S [M − H]^−^: 462.0265; found: 462.0284. Purity 99.8% by HPLC.

*N-(3,4-Dihydroxy-9,10-dioxo-9,10-dihydroanthracen-2-yl)-4-nitrobenzenesulfonamide* (**9b**). Orange solid, 50% yield. ^1^H-NMR (400 MHz, DMSO-*d*_6_) δ 12.61 (brs, 1H), 10.98 (brs, 1H), 10.79 (brs, 1H), 8.41 (d, *J* = 8.0 Hz, 2H), 8.25–8.06 (m, 4H), 7.97–7.86 (m, 2H), 7.73 (s, 1H). ^13^C-NMR (151 MHz, DMSO) δ 187.79, 180.53, 150.41, 149.90, 145.71, 143.61, 135.05, 134.24, 133.27, 132.79, 130.11, 128.22(2C), 126.77, 126.41, 124.65(2C), 123.70, 114.04, 113.50. MS (ESI) (*m*/*z)*: 439.0 (M − H)^−^. HRMS (ESI) calcd. for C_20_H_12_N_2_O_8_S [M − H]^−^: 439.0242; found: 439.0228. Purity 98.4% by HPLC.

*N-(3,4-Dihydroxy-9,10-dioxo-9,10-dihydroanthracen-2-yl)-4-(trifluoromethoxy) benzenesulfonamide* (**9c**). Yellow solid, 41% yield. ^1^H-NMR (400 MHz, DMSO-*d*_6_) δ 12.61 (brs, 1H), 10.94 (brs, 1H), 10.51 (brs, 1H), 8.22–8.12 (m, 2H), 8.05–7.99 (m, 2H), 7.95–7.85 (m, 2H), 7.74 (s, 1H), 7.61 (dd, *J* = 1.2, 9.2 Hz, 2H). ^13^C-NMR (151 MHz, DMSO) δ 187.78, 180.60, 151.20, 150.35, 142.96, 139.19, 135.02, 134.23, 133.30, 132.82, 130.63, 129.29, 126.78, 126.40, 123.74, 121.47, 119.80 (q, *J* = 259.7 Hz), 113.22, 113.12. MS (ESI) (*m*/*z*): 478.0 (M − H)^−^. HRMS (ESI) calcd. for C_21_H_12_F_3_NO_7_S [M − H]^−^: 478.0214; found: 478.0207. Purity 96.8% by HPLC.

*N-(3,4-Dihydroxy-9,10-dioxo-9,10-dihydroanthracen-2-yl)-2-(trifluoromethoxy) benzenesulfonamide* (**9d**). Yellow solid, 40% yield. ^1^H-NMR (400 MHz, DMSO-*d*_6_) δ 12.62 (brs, 1H), 11.00 (brs, 1H), 10.37 (brs, 1H), 8.24–8.09 (m, 2H), 8.00 (dd, *J* = 2.0, 8.0 Hz, 1H), 7.96–7.86 (m, 2H), 7.83–7.75 (m, 1H), 7.71 (s, 1H), 7.63–7.51 (m, 2H). ^13^C-NMR (151 MHz, DMSO) δ 187.81, 180.54, 150.37, 145.18, 143.20, 135.55, 135.05, 134.25, 133.28, 132.82, 132.34, 130.65, 130.41, 127.45, 126.80, 126.43, 123.61, 120.97, 119.81 (q, *J* = 259.7 Hz), 113.72, 113.26. MS (ESI) (*m*/*z*): 477.8 (M − H)^−^. HRMS (ESI) calcd. for C_21_H_12_F_3_NO_7_S [M − H]^−^: 478.0214; found: 478.0225. Purity 95.2% by HPLC.

*4-Cyano-N-(3,4-dihydroxy-9,10-dioxo-9,10-dihydroanthracen-2-yl) benzenesulfonamide* (**9e**). Red solid, 60% yield. ^1^H-NMR (400 MHz, DMSO-*d*_6_) δ 12.60 (brs, 1H), 10.77 (brs, 2H), 8.22–8.12 (m, 2H), 8.09 (d, *J* = 8.0 Hz, 2H), 8.02 (d, *J* = 8.4 Hz, 2H), 7.96–7.87 (m, 2H), 7.70 (s, 1H). ^13^C-NMR (151 MHz, DMSO) δ 187.81, 180.57, 150.39, 144.34, 143.46, 135.05, 134.25, 133.49, 133.29, 132.81, 130.22, 127.34, 126.81, 126.75, 126.44, 123.71, 117.60, 115.48, 113.78(2C), 113.44. MS (ESI) (*m*/*z*): 419.0 (M − H)^−^. HRMS (ESI) calcd. for C_21_H_12_N_2_O_6_S [M − H]^−^: 419.0343; found: 419.0348. Purity 95.1% by HPLC.

*N-(3,4-Dihydroxy-9,10-dioxo-9,10-dihydroanthracen-2-yl)-4-fluorobenzenesulfonamide* (**9f**). Yellow solid, 62% yield. ^1^H-NMR (400 MHz, DMSO-*d*_6_) δ 12.58 (brs, 1H), 10.84 (brs, 1H), 10.43 (brs, 1H), 8.19–8.11 (m, 2H), 8.01–7.84 (m, 4H), 7.73 (s, 1H), 7.46 (t, *J* = 8.8 Hz, 2H). ^13^C-NMR (151 MHz, DMSO) δ 187.75, 180.59, 164.47 (d, *J* = 250.7 Hz), 150.32, 142.76, 136.58, 134.99, 134.21, 133.28, 132.81, 130.82, 129.75 (d, *J* = 9.1 Hz,2C), 126.76, 126.38, 123.72, 116.57 (d, *J* = 22.7 Hz, 2C), 113.10, 112.89. MS (ESI) (*m*/*z*): 412.0 (M − H)^−^. HRMS (ESI) calcd. for C_20_H_12_FNO_6_S [M − H]^−^: 412.0297; found: 412.0305. Purity 98.7% by HPLC.

*N-(3,4-Dihydroxy-9,10-dioxo-9,10-dihydroanthracen-2-yl)-2-fluorobenzenesulfonamide* (**9g**). Yellow solid, 42% yield. ^1^H-NMR (400 MHz, DMSO-*d*_6_) δ 12.61 (brs, 1H), 10.95 (brs, 1H), 10.49 (brs, 1H), 8.21–8.09 (m, 2H), 7.95–7.80 (m, 3H), 7.77–7.67 (m, 2H), 7.46 (t, *J* = 9.4 Hz, 1H), 7.38 (t, *J* = 7.6 Hz, 1H). ^13^C-NMR (151 MHz, DMSO) δ 187.80, 180.56, 158.34 (d, *J* = 256.7 Hz), 150.40, 143.42, 136.02, 135.01, 134.21, 133.28, 132.81, 130.39, 129.87, 127.94 (d, *J* = 12.1 Hz), 126.77, 126.41, 124.90, 123.57, 117.44 (d, *J* = 21.1 Hz), 113.88, 113.32. MS (ESI) (*m*/*z*): 412.0 (M − H)^−^. HRMS (ESI) calcd. for C_20_H_12_FNO_6_S [M − H]^−^: 412.0297; found: 412.0300. Purity 96.1% by HPLC.

*N-(3,4-Dihydroxy-9,10-dioxo-9,10-dihydroanthracen-2-yl)-2,3-difluorobenzenesulfonamide* (**9h**). Red solid, 52% yield. ^1^H-NMR (400 MHz, DMSO-*d*_6_) δ 12.60 (brs, 1H), 10.83 (brs, 2H), 8.23–8.11 (m, 2H), 7.96–7.86 (m, 2H), 7.79 (q, *J* = 8.6 Hz, 1H), 7.69 (s, 1H), 7.62 (t, *J* = 7.0 Hz, 1H), 7.44–7.34 (m, 1H). ^13^C-NMR (151 MHz, DMSO) δ 187.86, 180.52, 150.46, 149.96 (dd, *J* = 249.9 Hz, J = 12.1 Hz), 146.64 (dd, *J* = 256.7 Hz, *J* = 13.6 Hz) 144.45, 135.05, 134.22, 133.29, 132.81, 130.25 (d, *J* = 10.6 Hz), 129.75, 126.78 (d, *J* = 10.6 Hz), 126.43 d, J = 7.6 Hz), 125.26, 124.95, 123.51, 122.86 (d, *J* = 16.6 Hz), 115.28 (d, *J* = 6.0 Hz), 113.72. MS (ESI) (*m*/*z*): 430.0 (M − H)^−^. HRMS (ESI) calcd. for C_20_H_11_FNO_6_S [M − H]^−^: 430.0202; found: 430.0206. Purity 95.4% by HPLC.

*4-Chloro-N-(3,4-dihydroxy-9,10-dioxo-9,10-dihydroanthracen-2-yl) benzenesulfonamide* (**9i**). Yellow solid, 56% yield. ^1^H-NMR (400 MHz, DMSO-*d*_6_) δ 12.61 (brs, 1H), 10.94 (brs, 1H), 10.47 (brs, 1H), 8.22–8.11 (m, 2H), 7.96–7.83 (m, 4H), 7.78–7.63 (m, 3H). ^13^C-NMR (151 MHz, DMSO) δ 187.76, 180.58, 150.34, 142.90, 139.07, 138.04, 135.00, 134.22, 133.28, 132.80, 130.63, 129.50 (2C), 128.57 (2C), 126.77, 126.39, 123.72, 113.18, 113.08. MS (ESI) (*m*/*z*): 427.8 (M − H)^−^. HRMS (ESI) calcd. for C_20_H_12_ClNO_6_S [M − H]^−^: 428.0001; found: 428.0005. Purity 99.4% by HPLC.

*2,4-Dichloro-N-(3,4-dihydroxy-9,10-dioxo-9,10-dihydroanthracen-2-yl) benzenesulfonamide* (**9j**). Orange solid, 60% yield. ^1^H-NMR (400 MHz, DMSO-*d*_6_) δ 12.62 (brs, 1H), 11.00 (brs, 1H), 10.56 (brs, 1H), 8.20–8.08 (m, 2H), 8.01 (d, *J* = 8.8 Hz, 1H), 7.95–7.84 (m, 3H), 7.68–7.63 (m, 2H). ^13^C-NMR (151 MHz, DMSO) δ 187.76, 180.50, 150.44, 143.33, 138.62, 136.55, 134.99, 134.20, 133.24, 132.79, 132.25, 132.12, 131.49, 130.24, 127.85, 126.75, 126.38, 123.63, 113.52, 113.36. MS (ESI) (*m*/*z*): 461.8 (M − H)^−^. HRMS (ESI) calcd. for C_20_H_11_Cl_2_NO_6_S [M − H]^−^: 461.9611; found: 461.9614. Purity 99.2% by HPLC.

*2,6-Dichloro-N-(3,4-dihydroxy-9,10-dioxo-9,10-dihydroanthracen-2-yl) benzenesulfonamide* (**9k**). Red solid, 12% yield. ^1^H-NMR (400 MHz, DMSO-*d*_6_) δ 12.62 (brs, 1H), 11.00 (brs, 1H), 10.56 (brs, 1H), 8.20–8.09 (m, 2H), 7.94–7.83 (m, 2H), 7.72–7.52 (m, 4H). ^13^C-NMR (151 MHz, DMSO) δ 187.71, 180.43, 150.40, 142.57, 134.95, 134.80, 134.29, 134.17, 134.13 (2C), 133.22, 132.78, 131.85 (2C), 130.41, 126.75, 126.36, 123.79, 113.20, 112.18. MS (ESI) (*m*/*z*): 461.8 (M − H)^−^. HRMS (ESI) calcd. for C_20_H_11_Cl_2_NO_6_S [M − H]^−^: 461.9611; found: 461.9601. Purity 95.7% by HPLC.

*2,4,6-Trichloro-N-(3,4-dihydroxy-9,10-dioxo-9,10-dihydroanthracen-2-yl) benzenesulfonamide* (**9l**). Yellow solid, 10% yield. ^1^H-NMR (400 MHz, DMSO-*d*_6_) δ 12.62 (brs, 1H), 10.96 (brs, 1H), 10.73 (brs, 1H), 8.21–8.11 (m, 2H), 7.96–7.86 (m, 4H), 7.66 (s, 1H). ^13^C-NMR (151 MHz, DMSO) δ 187.77, 180.49, 150.48, 143.28, 137.64, 135.25 (2C), 135.00, 134.43, 134.22, 133.27, 132.83, 131.20 (3C), 126.79, 126.41, 123.78, 113.43, 113.21. MS (ESI) (*m*/*z*): 495.8 (M − H)^−^. HRMS (ESI) calcd. for C_20_H_10_Cl_3_NO_6_S [M − H]^−^: 495.9222; found: 495.9220. Purity 99.4% by HPLC.

*4-Bromo-N-(3,4-dihydroxy-9,10-dioxo-9,10-dihydroanthracen-2-yl) benzenesulfonamide* (**9m**). Yellow solid, 65% yield. ^1^H-NMR (400 MHz, DMSO-*d*_6_) δ 12.60 (brs, 1H), 10.88 (brs, 1H), 10.47 (brs, 1H), 8.22–8.11 (m, 2H), 7.95–7.78 (m, 6H), 7.73 (s, 1H). ^13^C-NMR (151 MHz, DMSO) δ 187.77, 180.59, 150.33, 142.88, 139.50, 135.01, 134.23, 133.29, 132.81, 132.44 (2C), 130.65, 128.64 (2C), 127.08, 126.78, 126.39, 123.73, 113.18, 113.05. MS (ESI) (*m*/*z*): 471.8 (M − H)^−^. HRMS (ESI) calcd. for C_20_H_12_BrNO_6_S [M − H]^−^: 471.9496; found: 471.9495. Purity 96.0% by HPLC.

*2-Bromo-N-(3,4-dihydroxy-9,10-dioxo-9,10-dihydroanthracen-2-yl) benzenesulfonamide* (**9n**). Yellow solid, 46% yield. ^1^H-NMR (400 MHz, DMSO-*d*_6_) δ 12.63 (brs, 1H), 11.10 (brs, 1H), 10.24 (brs, 1H), 8.20–8.05 (m, 3H), 7.93–7.84 (m, 3H), 7.67–7.53 (m, 3H). ^13^C-NMR (151 MHz, DMSO) δ 187.72, 180.55, 150.37, 142.31, 138.96, 135.62, 134.97, 134.74, 134.21, 133.23, 132.80, 130.93, 130.76, 128.28, 126.75, 126.38, 123.75, 119.31, 113.07, 112.14. MS (ESI) (*m*/*z*): 471.8 (M − H)^−^. HRMS (ESI) calcd. for C_20_H_12_BrNO_6_S [M − H]^−^: 417.9496; found: 417.9498. Purity 97.5% by HPLC.

*3-Bromo-N-(3,4-dihydroxy-9,10-dioxo-9,10-dihydroanthracen-2-yl) benzenesulfonamide* (**9o**). Yellow solid, 54% yield. ^1^H-NMR (400 MHz, DMSO-*d*_6_) δ 12.60 (brs, 1H), 10.99 (brs, 1H), 10.53 (brs, 1H), 8.21–8.11 (m, 2H), 8.10 (s, 1H), 7.94–7.83 (m, 4H), 7.71 (s, 1H), 7.57 (t, *J* = 8.0 Hz, 1H). ^13^C-NMR (151 MHz, DMSO) δ 187.75, 180.55, 150.33, 142.90, 142.23, 136.02, 134.98, 134.19, 133.27, 132.79, 131.56, 130.49, 129.08, 126.76, 126.37, 125.61, 123.71, 122.16, 113.22, 112.97. MS (ESI) (*m*/*z*): 473.8 (M − H)^−^.HRMS (ESI) calcd. for C_20_H_12_BrNO_6_S [M − H]^−^: 417.9496; found: 417.9500. Purity 96.1% by HPLC.

*4-Iodo-N-(3,4-dihydroxy-9,10-dioxo-9,10-dihydroanthracen-2-yl) benzenesulfonamide* (**9p**). Yellow solid, 70% yield. ^1^H-NMR (400 MHz, DMSO-*d*_6_) δ 12.60 (brs, 1H), 10.87 (brs, 1H), 10.44 (brs, 1H), 8.23–8.10 (m, 2H), 7.99 (d, *J* = 8.0 Hz, 2H), 7.96–7.85 (m, 2H), 7.73 (s, 1H), 7.64 (d, *J* = 8.0 Hz, 2H). ^13^C-NMR (151 MHz, DMSO) δ 187.75, 180.61, 150.31, 142.73, 139.85, 138.23 (2C), 135.00, 134.22, 133.29, 132.82, 130.76, 128.27 (2C), 126.78, 126.39, 123.74, 113.12, 112.85, 101.56. MS (ESI) (*m*/*z*): 519.8 (M − H)^−^. HRMS (ESI) calcd. for C_20_H_12_INO_6_S [M − H]^−^: 519.9357; found: 519.9334. Purity 99.3% by HPLC.

*2-Iodo-N-(3,4-dihydroxy-9,10-dioxo-9,10-dihydroanthracen-2-yl) benzenesulfonamide* (**9q**). Yellow solid, 48% yield.^1^H-NMR (400 MHz, DMSO-*d*_6_) δ 12.64 (brs, 1H), 11.18 (brs, 1H), 10.02 (brs, 1H), 8.19–8.06 (m, 4H), 7.93–7.85 (m, 2H), 7.65 (s, 1H), 7.61 (td, *J* = 1.2, 8.0 Hz, 1H), 7.33 (td, *J* = 1.6, 8.0 Hz, 1H). ^13^C-NMR (151 MHz, DMSO) δ 187.72, 180.58, 150.30, 142.77, 142.13, 141.83, 134.97, 134.22, 134.21, 133.24, 132.81, 131.01, 130.16, 128.64, 126.76, 126.38, 123.83, 112.96, 111.59, 93.30. MS (ESI) (*m*/*z*): 519.8 (M − H)^−^. HRMS (ESI) calcd. for C_20_H_12_INO_6_S [M − H]^−^: 519.9357; found: 519.9356. Purity 96.3% by HPLC.

### 3.6. Synthesis of ethyl 2-((4-((4-chlorophenyl) sulfonamido)-1-hydroxy-9,10-dioxo-9,10-dihydroanthracen-2-yl) oxy) acetate *(**12**)*

Step 1. Compound **10** was synthesized according to the same route of compound **7** in which compound **5** was replaced by compound **6c**. Yellow solid, 73% yield.

Step 2. Compound **11** was synthesized according to the same route of compound **8** in which compound **7** was replaced by compound **10**. Purple solid, 93% yield.

Step 3. Compound **12** was synthesized according to the same route of **9i** in which compound **8** was replaced by compound **11**. Red solid, 6% yield. ^1^H-NMR (400 MHz, DMSO-d_6_) δ 13.37 (s, 1H), 12.44 (s, 1H), 8.20 (d, *J* = 7.2 Hz, 2H), 8.00–7.83 (m, 4H), 7.64 (d, *J* = 8.0 Hz, 2H), 7.32 (s, 1H), 5.09 (s, 2H), 4.25 (q, *J* = 7.2 Hz, 2H), 1.27 (t, *J* = 7.2 Hz, 3H).^13^C-NMR (151 MHz, DMSO) δ 187.87, 184.15, 167.48, 153.41, 150.30, 138.86, 137.31, 135.41, 135.23, 134.74, 133.52, 132.12, 129.90 (2C), 128.71 (2C), 127.06, 126.50, 114.75, 109.81, 107.99, 65.44, 61.24, 14.04. MS (ESI) (*m*/*z*): 514.2 (M − H)^−^. HRMS (ESI) calcd. for C_24_H_18_ClNO_8_S [M − H]^−^: 514.0369; found: 514.0364. Purity 96.0% by HPLC.

### 3.7. Synthesis of 2-((4-((4-chlorophenyl) sulfonamido)-1-hydroxy-9,10-dioxo-9,10-dihydroanthracen-2-yl) oxy) acetic acid *(**13**)*

Compound **13** was synthesized according to the same route of **6b** in which compound **6a** was replaced by compound **12.** Red solid, 95% yield. ^1^H-NMR (400 MHz, DMSO-d_6_) δ 13.36 (s, 1H), 12.53 (s, 1H), 8.23–8.13 (m, 2H), 7.98–7.84 (m, 4H), 7.64 (d, *J* = 8.4 Hz, 2H), 7.35 (s, 1H), 4.99 (s, 2H). ^13^C-NMR (151 MHz, Pyr) δ 188.75, 185.01, 171.15, 155.76, 152.45, 140.30, 139.12, 137.34, 134.82, 133.21, 130.44 (2C), 129.85 (2C), 127.85, 127.27, 121.12, 119.10, 115.52, 110.23, 108.94, 67.24. MS (ESI) (*m*/*z*): 485.8 (M − H)^−^. HRMS (ESI) calcd. for C_22_H_14_ClNO_8_S [M − H]^−^: 486.0056; found: 486.0061. Purity 98.0% by HPLC.

### 3.8. Synthesis of ethyl 2-((3-((4-chlorophenyl) sulfonamido)-1-hydroxy-9,10-dioxo-9,10-dihydroanthracen-2-yl) oxy) acetate *(**14a**)*

Compound **14a** was synthesized according to the same route of **6c** in which compound **5** was replaced by compound **9i**. Yellow solid, 24% yield. ^1^H-NMR (400 MHz, DMSO-*d*_6_) δ 12.74 (s, 1H), 10.59 (s, 1H), 8.22–8.09 (m, 2H), 8.01–7.87 (m, 4H), 7.83–7.68 (m, 3H), 4.85 (s, 2H), 4.19 (q, *J* = 7.2 Hz, 2H), 1.22 (t, *J* = 7.2 Hz, 3H). ^13^C-NMR (151 MHz, DMSO) δ 187.53, 180.83, 169.46, 154.03, 139.72, 138.49, 138.21, 136.81, 135.08, 134.58, 132.87, 132.72, 129.71 (2C), 128.70 (2C), 128.46, 126.88, 126.45, 113.29, 109.22, 68.53, 60.98, 13.98. MS (ESI) (*m*/*z*): 514.0 (M − H)^−^. HRMS (ESI) calcd. for C_24_H_18_ClNO_8_S [M − H]^−^: 514.0369; found: 514.0363. Purity 95.6% by HPLC.

### 3.9. Synthesis of 2-((3-((4-chlorophenyl) sulfonamido)-1-hydroxy-9,10-dioxo-9,10-dihydroanthracen-2-yl) oxy) acetic acid *(**14b**)*

Compound **14b** was synthesized according to the same route of **6b** in which compound **6a** was replaced by compound **14a**. Yellow solid, 95% yield. ^1^H-NMR (400 MHz, DMSO-*d*_6_) δ 12.72 (s, 1H), 8.19–8.10 (m, 2H), 7.97–7.87 (m, 4H), 7.75 (s, 1H), 7.71 (d, *J* = 8.4 Hz, 2H), 4.80 (s, 2H). ^13^C-NMR (151 MHz, DMSO) δ 187.49, 180.86, 171.92, 154.23, 139.93, 138.53, 138.14, 137.00, 135.06, 134.59, 132.86, 132.74, 129.76 (2C), 128.67 (2C), 128.58, 126.89, 126.45, 113.28, 108.86, 68.92. MS (ESI) (*m*/*z*): 486.0 (M − H)^−^. HRMS (ESI) calcd. for C_22_H_14_ClNO_8_S [M − H]^−^: 486.0056; found: 486.0049. Purity 99.0% by HPLC.

### 3.10. Synthesis of 4-chloro-N-(4-hydroxy-3-methoxy-9,10-dioxo-9,10-dihydroanthracen-2-yl) benzene- sulfonamide *(**14c**)*

Compound **9i** (215 mg, 0.5 mmol), K_2_CO_3_ (70 mg, 0.5 mmol) and iodomethane (70 mg, 12 mmol) were added to DMF (2.5 mL) and mixture was stirred at room temperature. The mixture was added to 10% aqueous HCl (20 mL). The suspension was extracted with ethyl acetate and the organic phase was washed with brine, dried over anhydrous Na_2_SO_4_ and filtered. The residue was purified by silica chromatography after removal of ethyl acetate to afford 14c as a yellow solid (115 mg, 52%). ^1^H-NMR (400 MHz, DMSO-*d*_6_) δ 12.68 (brs, 1H), 11.02 (brs, 1H), 8.28–8.14 (m, 2H), 7.99–7.89 (m, 2H), 7.70 (q, *J* = 8.5 Hz, 4H), 7.45 (s, 1H), 3.20 (s, 3H). ^13^C-NMR (151 MHz, DMSO) δ 188.19, 180.25, 151.50, 150.13, 138.19, 137.11, 135.30, 134.32, 133.37, 132.82, 132.10, 129.46 (2C), 129.16 (2C), 126.88, 126.62, 122.74, 121.75, 115.43, 37.30. MS (ESI) (*m*/*z*): 442.0 (M − H)^−^. HRMS (ESI) calcd. for C_21_H_14_ClNO_6_S [M − H]^−^: 442.0158; found: 442.0162. Purity 100% by HPLC. Purity 97.0% by HPLC.

### 3.11. Synthesis of 4-chloro-N-(4-hydroxy-3-methoxy-9,10-dioxo-9,10-dihydroanthracen-2-yl)-N-methyl- benzenesulfonamide *(**14d**)*

Compound **14d** was synthesized according to the same route of **14c** in which room temperature was raised to 40 °C. Orange solid, 50% yield. ^1^H-NMR (400 MHz, DMSO-*d*_6_) δ 12.79 (s, 1H), 8.28–8.13 (m, 2H), 8.00–7.92 (m, 2H), 7.76 (q, *J* = 8.4 Hz,4H), 7.40 (s, 1H), 3.80 (s, 3H), 3.23 (s, 3H). ^13^C-NMR (151 MHz, DMSO) δ 188.22, 180.56, 155.48, 150.66, 138.57, 138.37, 136.79, 135.40, 134.67, 132.96, 132.83, 129.62 (2C), 129.23 (2C), 127.10, 126.96, 126.72, 119.91, 116.60, 60.65, 38.12. MS (ESI) (*m*/*z*): 456.0 (M − H)^−^. HRMS (ESI) calcd. for C_22_H_16_ClNO_6_S [M − H]^−^: 456.0314; found: 456.0305. Purity 100% by HPLC.

### 3.12. Synthesis of 4-((4-chlorophenyl) sulfonyl)-12-hydroxy-3,4-dihydro-2H-anthra[2,3-b][1,4]-oxazine- 6,11-dione *(**14e**)*

Compound **14e** was synthesized according to the same route of **14c** in which iodomethane was replaced by 1,2-dibromoethane. Orange solid, 32% yield. ^1^H-NMR (400 MHz, DMSO-*d*_6_) δ 12.63 (s, 1H), 8.18 (t, *J* = 8.2 Hz, 2H), 8.11 (s, 1H), 7.98–7.88 (m, 2H), 7.84 (d, *J* = 8.4 Hz, 2H), 7.72 (d, *J* = 8.4 Hz, 2H), 4.06 (dd, *J* = 4.4, 16.8 Hz, 4H). ^13^C-NMR (151 MHz, DMSO) δ 187.52, 180.70, 151.78, 140.17, 139.33, 136.21, 135.07, 134.43, 133.27, 132.79, 130.21 (2C), 129.30, 129.04 (2C), 126.92, 126.55, 124.47, 113.41, 112.82, 64.03, 43.77. MS (ESI) (*m*/*z*): 456.0 (M + H)^+^. HRMS (ESI) calcd. for C_22_H_14_ClNO_6_S [M + H]^+^: 456.0303; found: 456.0307. Purity 100% by HPLC.

### 3.13. Synthesis of 4-chloro-N-(3-(2-hydrazinyl-2-oxoethoxy)-4-hydroxy-9,10-dioxo-9,10-dihydroanthracen-2-yl) benzenesulfonamide *(**14f**)*

Compound **14a** (20 mg, 0.04 mmol) and hydrazine hydrate (85%, 5 μL, 0.16 mmol) were added to ethanol (1.5 mL) and mixture was stirred at 76 °C. A yellow solid (14 mg, 72%) of **14f** was obtained by filtration. ^1^H-NMR (400 MHz, DMSO-*d*_6_) δ 12.95 (brs, 1H), 8.43–7.36 (m, 9H), 4.53 (s, 2H). ^13^C-NMR (151 MHz, DMSO) δ 187.43, 180.96, 168.15, 154.79, 140.49, 138.43, 135.10, 134.98, 134.67, 132.89, 132.81, 129.78 (2C), 129.08, 128.64 (2C), 128.51, 126.95, 126.49, 113.41, 109.56, 70.47. MS (ESI) (*m*/*z*): 500.0 (M − H)^−^. HRMS (ESI) calcd. for C_22_H_16_ClN_3_O_7_S [M − H]^−^: 500.0325; found: 500.0340. Purity 95.1% by HPLC.

### 3.14. Synthesis of 3-((4-chlorophenyl) sulfonamido)-1-hydroxy-9,10-dioxo-9,10-dihydroanthracen-2-yl methanesulfonate *(**14g**)*

Compound **9i** (107 mg, 0.25 mmol) and DIPEA (50 μL, 0.3mmol) were added to pyridine (1.5 mL), and then methanesulfonyl chloride (23 μL,0.3 mmol) was added to the mixture. After being stirred at room temperature for 4 h, the mixture was added to 10% aqueous HCl (20 mL). The suspension was extracted with ethyl acetate and the organic phase was washed with brine, dried over anhydrous Na_2_SO_4_ and filtered. The residue was purified by silica chromatography after removal of ethyl acetate to afford 14 g as a yellow solid (83 mg, 65%). ^1^H-NMR (400 MHz, DMSO-*d*_6_) δ 12.81 (s, 1H), 8.23–8.11 (m, 2H), 8.00–7.90 (m, 4H), 7.72 (d, *J* = 8.4 Hz, 2H), 7.64 (s, 1H), 3.61 (s, 3H). ^13^C-NMR (151 MHz, DMSO) δ 186.86, 180.93, 155.49, 138.86, 138.32, 135.07, 134.76, 132.83 (2C), 131.81, 131.31, 129.70 (3C), 128.79, (3C) 126.98, 126.50, 109.55, 40.45. MS (ESI) (*m*/*z*): 505.8 (M − H)^−^. HRMS (ESI) calcd. for C_21_H_14_ClNO_8_S_2_ [M − H]^−^: 505.9777; found: 505.9791. Purity 99.5% by HPLC.

### 3.15. PGAM1 Enzymatic Assay

Firstly, 1 μL inhibitor in dimethyl sulfoxide (DMSO) incubated with 49 μL 4.6 nM recombinant PGAM1, then 50 μL enzyme mix containing 3 units/mL enolase (Sigma-Aldrich, Saint Louis, MO, USA), 3 units/mL recombinant pyruvate kinase M2 (Sigma-Aldrich), 0.6 units/mL recombinant lactatdehydrogenase (LDH, Sigma-Aldrich), 100 mM Tris-HCl, 100 mM KCl, 5 mM MgCl_2_, 1 mM adenosine diphosphate (ADP), 0.2 mM the reduced form of nicotinamide adenine dinucleotide (NADH) and 4 mM 3PG was added. PGAM1 activity was measured as the decrease in OD at 340 nm. In addition, we performed a counter screening in which 4mM 2PG was added to 50 μL reaction mix containing the indicated inhibitor, 3 units/mL enolase (Sigma-Aldrich), 3 units/mL recombinant pyruvate kinase M2 (Sigma-Aldrich), 0.6 units/mL recombinant LDH (Sigma-Aldrich), 100 mM Tris-HCl, 100 mM KCl, 5 mM MgCl_2_, 1 mM ADP and 0.2 mM NADH. The other three enzyme activity was measured as the decrease in OD at 340 nm [17].

### 3.16. Constructions of Mutations of PGAM1

The wild-type plasmid was a gift from Department of Chemistry and Institute for Biophysical Dynamics, University of Chicago (Chicago, IL, USA). To obtain PGAM1 F22A/R116H/R191H mutant construct, the quick change kit (#KOD-401, TOYOBO, Osaka, Japan) was used to make site-specific mutagenesis, which confirmed by standard DNA sequencing methods. The primers used for the mutation were listed below:

PGAM1 F22A Forward: GAACCTGGAGAACCGCGCCAGCGGCTGGTACGAC
PGAM1 F22A Reverse: GTCGTACCAGCCGCTGGCGCGGTTCTCCAGGTTC
PGAM1 K116H Forward: CAGGTGAAGATCTGGCACCGCTCCTATGATGTCC
PGAM1 K116H Reverse: GGACATCATAGGAGCGGTGCCAGATCTTCATCTG
PGAM1 K119H Forward: CATGGCAACAGCCTCCACGGCATTGTCAAGCAT
PGAM1 K119H Reverse: ATGCTTGACAATGCCGTGGAGGCTGTTGCCATG

### 3.17. Protein Purification, Crystallization, Data Collection and Structure Determination

The C-terminal His_6_-tagged PGAM1 was expressed and affinity purified following the reported protocols [22]. The crystals of PGAM1 were obtained using the hanging drop vapor-diffusion method at 16 °C in a crystallization buffer containing of 8% (*w*/*v*) PEG3350 and 100 mM MES 6.0. To obtain the co-crystal of PGAM1 with compound **9i**, the crystals of PGAM1 were soaked in stock solution containing 500 μM **9i** for 2 h. Crystals were then cryo-protected by brief soaking in a mixture solution of crystallization buffer:glycerol (76:24) and quickly cooled in liquid nitrogen. Diffraction data were collected at beamline BL17U1, BL18U1 and BL19U1 in the Shanghai Synchrotron Radiation Facility (SSRF, Shanghai, China) using an X-ray beam of wavelength 0.97851 Å. The data was handled with HKL3000 [41] and the structure was determined by using molecular replacement in CCP4 [42] via an initial model of PGAM1 derived from PDB entry 4GPZ [22]. The model was then refined to 1.98 Å resolution using Phenix. The ligand restraints were generated using eLBOW in Phenix and manual rebuilding of the model was completed using the molecular graphics program COOT [43] according to the electronic density. All the graphs were plotted by Pymol (DeLano Scientific LLC, San Carlos, CA, USA).

### 3.18. Cell Viability Assays

The H1299 cell line was obtained from Guangzhou Jenniobio Biotechnology Co., Ltd. (Guangzhou, China), PC9 and A549 cells were gifted from Deng Jiong’s lab in the School of Medicine, Shanghai Jiao Tong University, Shanghai, China). H1299, PC9 cells were cultured in RPMI-1640 medium and A549 cells were cultured in F-12K medium containing 10% fetal bovine serum (FBS), 100 units/mL of Penicillin and 100 μg/mL Streptomycin. In cell viability assays, 2000 H1299 cells, A549 cells or 1000 PC9 cells per well were seeded in 96-well plate. After attachment for 24 h, the cells were treated with indicated inhibitor for 72 h. After incubation with 0.5 mg/mL methylthiazolyldiphenyl-tetrazolium bromide (MTT) for 4 h, cell viability was measured as the OD at 570 nm.

## 4. Conclusions

In summary, we designed and synthesized 31 anthraquinone derivatives as PGAM1 inhibitors. The SAR study focused on the effect of 1,2-substituents, 3-substituents, 2,4-substituents, and 2,3-substituents on the anthraquinone core scaffold activity against PGAM1. Among them, 3- sulfonamide substituents but not 4-substituents on the anthraquinone scaffold were essential for maintaining potency, which was further revealed by the X-ray structure of PGAM1 complexed with compound **9i** with IC_50_ value of 0.27 μM. In addition, modifications of the hydroxyl of alizarin disrupted the π-cation interaction of PGAM1 and the anthraquinone derivatives which led to the loss of potency. Taken as a whole, the SAR study of anthraquinone derivatives against PGAM1 provided useful information for further discovery of PGAM1 inhibitors.

## Figures and Tables

**Table 1:**
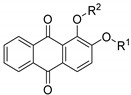
Inhibition activity of compounds **6a**–**e** towards PGAM1**.**

Compound	R ^1^	R ^2^	Inhibition of PGAM1 Activity at 10 μM ^1^
**6a**	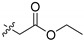	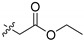	−0.6 ± 1%
**6b**	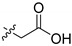	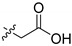	14.3 ± 5.9%
**6c**	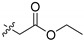	H	0 ± 1.8%
**6d**	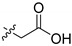	H	5.5 ± 0.03%
**6e**	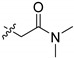	H	20.2 ± 2.2%

^1^ The data are presented as mean ±s.d. (n = 2).

**Table 2:**
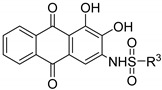
Inhibition activity of compounds **9a**–**q** towards PGAM1.

Compound	R ^3^	PGAM1 IC_50_ (μM) ^1^
**9a**	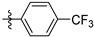	0.54 ± 0.04
**9b**	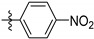	0.47 ± 0.08
**9c**	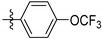	0.90 ± 0.07
**9d**	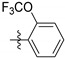	2.2 ± 0.5
**9e**	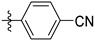	0.61 ± 0.12
**9f**	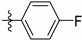	0.54 ± 0.07
**9g**	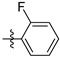	0.79 ± 0.04
**9h**	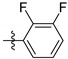	0.89 ± 0.13
**9i**	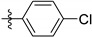	0.27 ± 0.04
**9j**	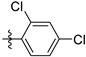	0.28 ± 0.07
**9k**	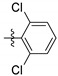	0.89 ± 0.33
**9l**	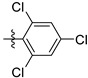	0.90 ± 0.26
**9m**	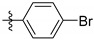	0.26 ± 0.02
**9n**	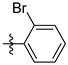	0.20 ± 0.04
**9o**	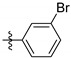	0.35 ± 0.005
**9p**	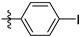	0.47 ± 0.07
**9q**	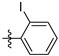	0.26 ± 0.03

^1^ The data are presented as mean ± s.d. (n = 3).

**Table 3:**
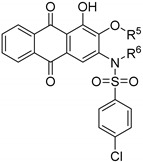
Inhibition activity of compounds **14a**–**14g** towards PGAM1.

Compound	R ^5^	R ^6^	PGAM1 IC_50_ (μM) ^1^
**14a**	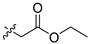	H	>10 μM
**14b**	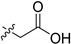	H	>10 μM
**14c**	CH_3_	H	2.92 ± 0.11
**14d**	CH_3_	CH_3_	7.01 ± 0.92
**14e**	-CH_2_-	-CH_2_-	>10 μM
**14f**	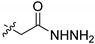	H	2.0 ± 0.2
**14g**	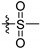	H	>10 μM

^1^ The data are presented as mean ±s.d. (n = 3).

**Table 4:** The inhibition in the control linked enzyme assay of selected compounds.

Compound	PGAM1 IC_50_ (μM)	Inhibition in the Control Linked Enzyme Assay (0.5 μM) ^1^
**9a**	0.54 ± 0.04	8.0 ± 5.0%
**9b**	0.47 ± 0.08	−2.9 ± 0.3%
**9c**	0.90 ± 0.07	3.7 ± 1.8%
**9e**	0.61 ± 0.12	6.7 ± 0.2%
**9f**	0.54 ± 0.07	5.7 ± 0.7%
**9g**	0.79 ± 0.04	10.2 ± 3.0%
**9h**	0.89 ± 0.13	0.06 ± 0.4%
**9i**	0.27 ± 0.04	−6.4 ± 5.0%
**9j**	0.28 ± 0.07	−2.0 ± 5.1%
**9k**	0.89 ± 0.33	0.5 ± 3.2%
**9l**	0.90 ± 0.26	0.2 ± 1.7%
**9m**	0.26 ± 0.02	−0.1 ± 2.5%
**9n**	0.20 ± 0.04	6.1 ± 0.8%
**9o**	0.35 ± 0.005	7.7 ± 0.2%
**9p**	0.47 ± 0.07	1.9 ± 0.1%
**9q**	0.26 ± 0.03	−2.3 ± 2.4%

^1^ The data are presented as mean ±s.d. (n = 2).

**Table 5:** Data collection and refinement statistics.

Property	Value
Data Collection	
Space group	P 2_1_ 2_1_ 2_1_
Cell constants a, b, c (Å)	82.54, 82.76, 104.20
α, β, γ (°)	90.00, 90.00, 90.00
Resolution *(Å)	50–1.98 (2.05–1.98)
% Data completeness	100.0 (100.0)
No. Observations	330639
No. Unique Reflections	50593 (4986)
Redundancy	6.5 (6.6)
R_merge_	0.06 (0.88)
< I/σ(I) >	2.05 (at 1.98Å)
CC1/2	0.996 (0.728)
**Refinement**	
Resolution (Å)	38.89–1.98 (2.02–1.98)
% Data completeness	100.0 (100.0)
No. reflections	50362 (2636)
R_work_/R_free_	0.197/0.225
R_free_ test set	2569 reflections (5.08%)
Wilson B-factor (Å^2^)	39.4
F_o_, F_c_ correlation	0.96
Total number of atoms	4046
Average B, all atoms (Å^2^)	41.0
Ramachandran favored (%)	98.7
Ramachandran allowed (%)	1.3
Ramachandran outliers (%)	0.0
Rmsd bond lengths (Å)	0.007
Rmsd bond angles (°)	1.044
**PDB Accession Code**	6ISN

**Table 6:**
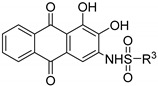
Inhibition activity of compounds **9a**–**q** on cancer cell proliferation.

Compound	R^3^	H1299 IC_50_ (μM)	A549 IC_50_ (μM)	PC9 IC_50_ (μM) ^1^
**9a**	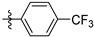	20.7 ± 0.6	n.d.	n.d.
**9b**	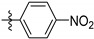	25.8 ± 2.2	n.d.	n.d.
**9c**	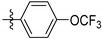	16.6 ± 1.8	8.0 ± 0.8	10.2 ± 0.7
**9d**	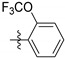	13.9 ± 3.6	17.0 ± 4.9	44.2 ± 1.5
**9e**	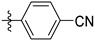	26.3 ± 4.5	n.d.	n.d.
**9f**	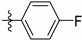	24.2 ± 2.6	n.d.	n.d.
**9g**	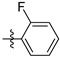	31.3 ± 1.9	n.d.	n.d.
**9h**	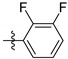	45.7 ± 2.7	n.d.	n.d.
**9i**	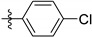	6.9 ± 1.2	12.7 ± 2.7	13.8 ± 1.0
**9j**	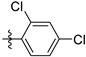	23.7 ± 1.1	n.d.	n.d.
**9k**	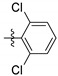	34.6 ± 2.4	n.d.	n.d.
**9l**	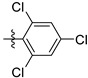	6.2 ± 1.2	>50	>50
**9m**	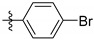	28.1 ± 1.9	n.d.	n.d.
**9n**	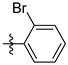	50 ± 0.6	n.d.	n.d.
**9o**	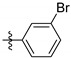	24.5 ± 0.2	n.d.	n.d.
**9p**	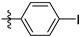	17.3 ± 3.9	10.3 ± 1.4	13.4 ± 1.8
**9q**	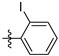	26.4 ± 3.8	n.d.	n.d.

^1^ The data are presented as mean ±s.d. (n = 3) n.d. = not determined.

**Table 7:**
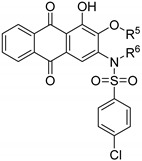
Inhibition activity of compounds **14a**–**g** on cancer cell proliferation.

Compound	R^5^	R^6^	H1299 IC_50_ (μM)	A549 IC_50_ (μM)	PC9 IC_50_ (μM) ^1^
**14c**	CH_3_	H	26.9 ± 2.1	n.d.	n.d.
**14d**	CH_3_	CH_3_	10.3 ± 0.8	9.9 ± 0.2	49.1 ± 1.9
**14f**	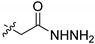	H	>100	n.d.	n.d.
**14g**	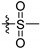	H	28.5 ± 0.6	n.d.	n.d.

^1^ The data are presented as mean ±s.d. (n = 3) n.d. = not determined.

## Supplementary Materials

The following are available online, Figure S1: The activity of PGAM1 mutant.
