# Supplementary material for: Development of Anthraquinone Analogues as Phosphoglycerate Mutase 1 Inhibitors
Source: Molecules. 2019 Feb 27;24(5):845. doi: 10.3390/molecules24050845 (PMC6429356; doi:10.3390/molecules24050845)
Supplement: Supplementary file 1 [file molecules-24-00845-s001.pdf]

molecules

Supplementary Data

Development of Anthraquinone Analogues as Phosphoglycerate Mutase 1 Inhibitors

Ke Huang, Lulu Jiang, Huiti Li, Deyong Ye * and Lu Zhou *

Department of Medicinal Chemistry, School of Pharmacy, Fudan University, No. 826, Zhangheng Rd., Shanghai 201203, China; kehuang13@fudan.edu.cn (K.H.); 18111030012@fudan.edu.cn (L.J.); 17211030005@fudan.edu.cn (H.L.)

* Correspondence: dyye@shmu.edu.cn (D.Y.); zhoulu@fudan.edu.cn (L.Z.); Tel.: +86-21-5198-0117 (D.Y.); +86-21-5198-0125 (L.Z.)

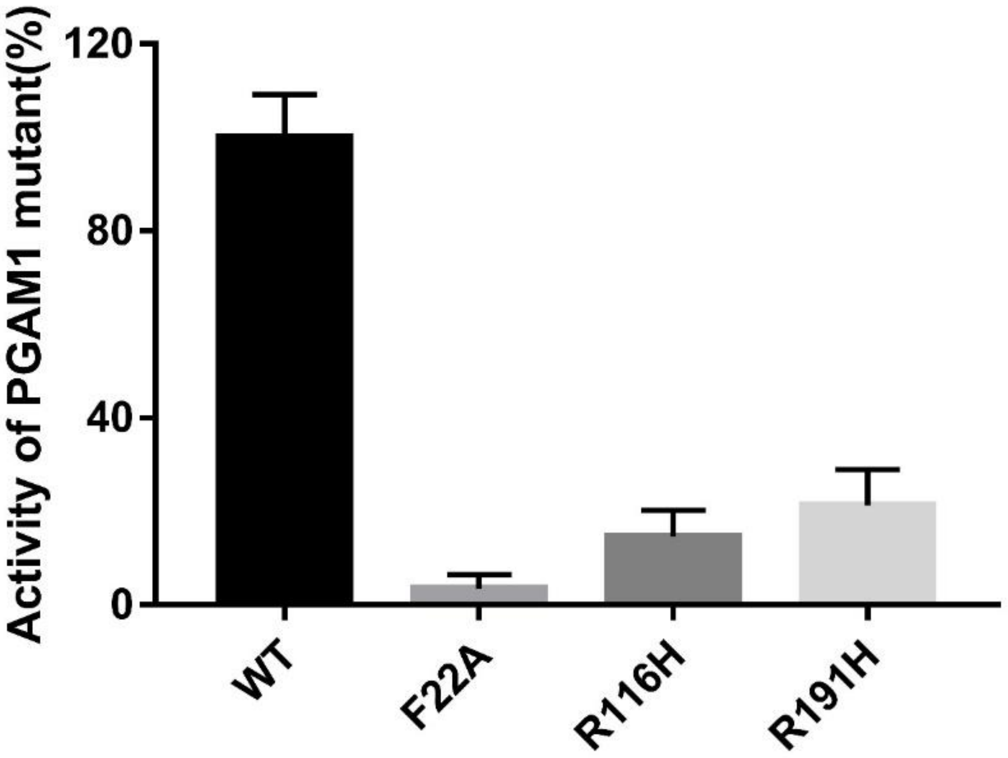

Figure S1. The activity of PGAM1 mutant.
